# Visual response of ventrolateral prefrontal neurons and their behavior-related modulation

**DOI:** 10.1038/s41598-021-89500-0

**Published:** 2021-05-12

**Authors:** Stefano Rozzi, Marco Bimbi, Alfonso Gravante, Luciano Simone, Leonardo Fogassi

**Affiliations:** 1 Department of Medicine and Surgery, University of Parma, via Volturno 39, 43125 Parma, Italy; 2 Institut des Sciences Cognitives Marc Jeannerod, CNRS, Université de Lyon, 67 Boulevard Pinel, 69500 Bron, France; 3 Cognition, Motion and Neuroscience, Center for Human Technologies, Istituto Italiano di Tecnologia, Genoa, Italy

**Keywords:** Cognitive neuroscience, Motor control, Sensorimotor processing, Sensory processing

## Abstract

The ventral part of lateral prefrontal cortex (VLPF) of the monkey receives strong visual input, mainly from inferotemporal cortex. It has been shown that VLPF neurons can show visual responses during paradigms requiring to associate arbitrary visual cues to behavioral reactions. Further studies showed that there are also VLPF neurons responding to the presentation of specific visual stimuli, such as objects and faces. However, it is largely unknown whether VLPF neurons respond and differentiate between stimuli belonging to different categories, also in absence of a specific requirement to actively categorize or to exploit these stimuli for choosing a given behavior. The first aim of the present study is to evaluate and map the responses of neurons of a large sector of VLPF to a wide set of visual stimuli when monkeys simply observe them. Recent studies showed that visual responses to objects are also present in VLPF neurons coding action execution, when they are the target of the action. Thus, the second aim of the present study is to compare the visual responses of VLPF neurons when the same objects are simply observed or when they become the target of a grasping action. Our results indicate that: (1) part of VLPF visually responsive neurons respond specifically to one stimulus or to a small set of stimuli, but there is no indication of a “passive” categorical coding; (2) VLPF neuronal visual responses to objects are often modulated by the task conditions in which the object is observed, with the strongest response when the object is target of an action. These data indicate that VLPF performs an early passive description of several types of visual stimuli, that can then be used for organizing and planning behavior. This could explain the modulation of visual response both in associative learning and in natural behavior.

## Introduction

In the lateral prefrontal cortex (LPF) there are many neurons responding to visual stimuli. These visual responses have been generally interpreted in terms of categorical coding or as the result of an association with subsequent behavioral reactions^1–7^. In the latter case, visual responses are often followed by a memory-related sustained activity^8–12^.

It is generally accepted that visual responses of prefrontal neurons can be related either to object features or to spatial characteristics. This distinction would correspond to a subdivision of LPF into two functional sectors: a dorsal part (dorsolateral prefrontal cortex, DLPF) mainly involved in the elaboration of spatial aspects of visual information, and a ventral part (ventrolateral prefrontal cortex, VLPF) more related to the analysis of objects features^12–14^. This subdivision is in line with neuroanatomical data, showing that the dorsal part is mainly connected to posterior parietal cortex, while the ventral part with inferotemporal areas^15–19^. However, this sharp dichotomy has been challenged by a series of electrophysiological studies using visual stimuli differing in spatial location, shape and color^20,21^. These authors confirm that DLPF shows a higher selectivity for spatial location, while their studies do not clearly support a strong preference of VLPF for objects with respect to spatial features. An exception to this finding would be represented only by very specific visual stimuli, such as faces, that are usually reported to activate distinct patches of VLPF^22^. In addition, space and object specificity appears more pronounced in the posterior sectors of both DLPF and VLPF, since going more rostrally the neural responses become more abstract and more tuned to the characteristics of the behavioral task^20^ (for a similar view in humans, see^23,24^). In line with this redefinition of the dichotomy, it stands the neuroanatomical evidence that VLPF, besides the inferotemporal afference, receives also a strong parietal input functionally related to eye, arm/hand and mouth fields^17,18,25–30^.

Previous studies on visual responses in LPF mainly focused on the manipulation of visual input for guiding behavior, with paradigms requiring monkeys to learn an arbitrary association between a specific visual stimulus and, for example, a saccade or a reaching movement^5,31–33^, see^3,8,34^. Another series of studies assessed the role of prefrontal neurons in the categorization of visual stimuli, for example requiring the monkey to actively discriminate between objects belonging to different categories^1,7,35^. On the contrary, few studies investigated the neuronal responses to passive presentation of visual stimuli^20,21,36^, and many specifically focused on visual responses to faces, mainly in a restricted sector of caudal VLPF^37–40^.

Thus, the *first aim* of this study was to evaluate and map the responses of neurons of a large sector of VLPF to a wide set of visual stimuli, with the only requirement for the monkey to fixate the image, without using it for a specific behavior. To achieve this aim, we recorded the neuronal activity from VLPF of two monkeys while they observed static images presented on a monitor (Visual task). In order to assess whether prefrontal neurons categorize visual stimuli even in the absence of a specific instruction we chose stimuli belonging to four semantic macro-categories.

Previous studies demonstrated the presence of VLPF neurons responding when monkeys observed real objects that represented the targets of grasping actions and also during the actual grasping of these same object. This suggested a link between the visual and motor activation^41,42^. On the basis of these results, the *second aim* of this study is to verify whether prefrontal neurons (1) respond differently to pictures of graspable objects with respect to the same, real, objects; (2) have a different activation when objects are passively observed or become target of a grasping action. To this purpose, we analyzed the neuronal responses recorded during a visuo-motor task, in which the monkeys had to observe real objects and, instructed by a cue, to grasp them or refrain from acting. These responses have been then compared with those recorded, in the same neurons, during the visual task.

## Materials and methods

### Subjects

The experiment was carried out on two female Rhesus monkeys (*Macaca mulatta*, M1, M2) weighing about 4 kg. The animals have been previously employed in a series of experiments, whose results have been published^41,43^. All methods were carried out in accordance with the European (2010/63/EU) and the ARRIVE guidelines. The experimental protocols, the animal handling, and the surgical and experimental procedures, complied with the European guidelines (2010/63/EU), and Italian laws in force on the care and use of laboratory animals, and were approved by the Veterinarian Animal Care and Use Committee of the University of Parma (Prot. 78/12, 17/07/2012; Prot. 91/OPBA/2015, 21/10/2015) and authorized by the Italian Health Ministry (D.M. 294/2012-C, 11/12/2012; 48/2016-PR, 20/01/2016).

### Training and surgical procedures

The monkeys were first trained to seat on a primate chair and to familiarize with the experimental setup. At the end of the habituation sessions, a head fixation system (Crist Instruments Co. Inc.) was implanted. Then, they were trained to perform the visual tasks described below. After completion of the training, a recording chamber (32 × 18 mm, Alpha Omega, Nazareth, Israel) was implanted on VLPF, based on MRI scan. All surgeries were carried out under general anesthesia (ketamine hydrocloride, 5 mg/kg, i.m. and medetomidine hydrocloride, 0.1 mg/kg, i.m.), followed by postsurgical pain medication^25,41,43,44^.

### Recording techniques and signal acquisition

Single unit recording was performed using a multi-electrode recording system (AlphaLab Pro, Alpha Omega Engineering, Nazareth, Israel). The microelectrodes, glass-coated (impedance, 0.5–1 MΩ), were mounted on an electrode holder (MT, Microdriving Terminal, Alpha Omega) that, by means of dedicated engines, controlled by a software (EPS; Alpha Omega), allowed electrodes vertical displacement. The MT holder was directly fixed to the recording chamber. Neuronal activity was filtered, amplified and monitored with a multichannel processor and sorted using a multi-spike detector (MCP Plus 8 and ASD, Alpha Omega Engineering). Spike sorting was performed using an Off-line Sorter (Plexon, Inc, Dallas TX, USA).

The experiment was controlled by a homemade Labview software. The digital signals provided time-related information of task phases (the onset and offset of fixation point, stimuli presentation and reward delivery) and behavioral events (monkey hand contact with the starting point, beginning and end of object pulling) and were then used for aligning the neural activity.

Analog signals provided information about eye position. Eye movements were recorded at 120 Hz using an infrared pupil/corneal reflection tracking system (Iscan Inc., Cambridge, MA, USA) positioned above the screen in front of the monkey.

### Experimental apparatus

During training and recording sessions the monkeys seated on the monkey chair with the hand contralateral to the hemisphere to be recorded on a resting position. The monitor where visual stimuli were presented in the Visual task (see below) was positioned at 54 cm from monkey’s eyes. Monitor resolution was of 1680 × 1050 pixel and its geometrical center was located at the level of monkey eyes. A laser spot could be projected on the center of the screen as fixation point. A phototransistor was placed on the monitor in order to provide the onset and offset of the visual stimuli.

A box containing three objects was placed at 22 cm from the monkey’s chest during the Visuo-Motor task (see below). The opening of a small door (7 × 7 cm) facing the monkey at eye height allowed to present three objects, one at the time. Two laser spots (instructing cues) of different colors (green and red) were projected onto the box door or onto the object, signaling the task conditions and phases.

### Behavioral paradigms and stimuli

#### Visual task

**Figure 1:**
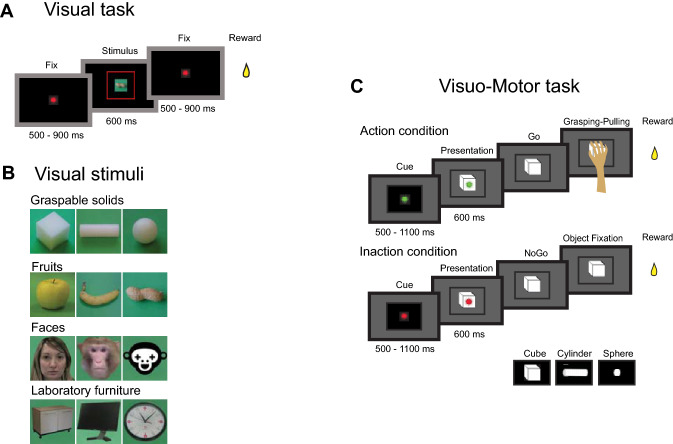
(**A**) Temporal sequence of events in the Visual task; (**B**) stimuli presented in the visual task; **C** Temporal sequence of events in the Visuo-Motor task.

To evaluate the response of VLPF neurons to observation of visual stimuli, we displayed images (6° × 6°) depicting 12 stimuli (see below), while the monkeys had to keep their gaze within stimulus limits. Figure 1A shows the sequence of events occurring during each trial. The monkeys were required to keep their hand on the resting position; if this was accomplished, the trial started, and the fixation point was turned on. They were required to fixate it for a randomized time interval (500–900 ms), keeping the eye within a ± 3° (X and Y) fixation window. If they kept fixation for this period of time, the fixation point turned off and one of the images was presented for 600 ms. The monkeys had to observe it throughout the presentation period, keeping fixation within the fixation window. Then, the image disappeared, the fixation point turned on again for a randomized period (500–900 ms) and the monkeys had to keep fixation on it. The trials were accepted as correct, and the monkeys were rewarded, if they kept their eyes within the fixation window for the duration of each phase of the task (first fixation, stimulus presentation and second fixation) and did not release the hand from the resting position. Discarded trials were repeated at the end of the sequence to collect at least 10 presentations for each stimulus. The order of stimuli presentation was randomized.

The 12 stimuli (Fig. 1B) belong to 4 different semantic categories:

Graspable solids (pictures of the objects employed in the motor task described in Simone et al.^41^): cube, cylinder, sphere;

- Fruits: apple, banana, peanut;
- Faces: human face, monkey face, sketchy drawing of a face;
- Laboratory furniture, geometric, but not graspable: shelf, monitor and clock.

The stimuli had a similar luminance and were presented on a homogeneous green background. Note that the fruits and solids could evoke similar affordances (apple and cube: power grip; banana and cylinder: finger prehension; sphere and peanut: precision grip).

Informed consent to publish identifying images (human face) has been obtained.

#### Visuo-Motor task

The Visuo-Motor task corresponds to that described in^41^. Briefly, the task consisted of two basic conditions: Action and Inaction (Fig. 1C). Each trial started with the monkeys’ hand on a starting position. Then one of the two instructing cues (green = Action condition; red = Inaction condition) turned on and projected onto the closed box door. In both conditions, the monkeys had to maintain fixation within a 6° × 6° fixation window centered on the instructing cue for a randomized time interval (500–1100 ms). Then the box door opened allowing the monkey to see one of three objects. The objects were a small sphere (diameter 1 cm), a large cube (side 2 cm) and a cylinder (length 4 cm, diameter 1.5 cm, horizontally oriented).

In the Action condition, during object presentation, the monkeys had to maintain fixation and the green cue was still on, projected onto the object. After a randomized time (700 to 1100 ms) the green cue turned off (Go signal), instructing the monkeys to reach for, grasp the object and pull it. If the monkeys correctly performed a trial, a drop of liquid reward was delivered at the end of it.

In the Inaction condition, the monkeys were instructed by a red cue whose extinction required the monkeys to keep fixating the object for 600 ms. After correct completion of the trial, the monkey was rewarded as in the Action condition. The order of presentation of both objects and conditions was randomized.

### Data analysis

#### Visual task

In the visual task, we recorded neural activity for at least 120 successful trials, 10 for each stimulus. For the statistical analysis, two epochs were defined: (1) *Baseline*: 500 ms preceding stimulus presentation, during which the monkey was looking at the fixation point; (2) *Stimulus*: the first 500 ms of image presentation.

Single-neuron responses were statistically evaluated by means of a 2X12 ANOVA for repeated measures (Factors: Epochs, Stimuli, *p* < 0.01) followed by Newman-Keuls post hoc tests.

A neuron was considered visually responsive when the 2X12 ANOVA revealed: (1) a significant Main effect Epoch and/or (2) a significant Interaction effect, in which the Post-hoc test showed a significant difference between at least one stimulus epoch of one image and its baseline.

Visually responsive neurons were classified as selective when the 2X12 ANOVA revealed a significant Interaction effect and the post-hoc test showed a significant difference among the activity recorded in the stimulus epoch of one stimulus and that of (1) its baseline and (2) the stimulus epoch of at least another stimulus. Neurons were classified as unselective when the statistical test revealed a significant Main effect Epoch and/or a significant Interaction effect, and the post-hoc test did not show any difference among the activities recorded in the stimulus epoch of the 12 stimuli.

#### Comparison of neuron response in the Visual and Visuo-Motor tasks

In the Visuo-Motor task, the neural activity was recorded for at least 60 successful trials (30 per condition, 10 for each object). For statistical analysis of the neural activity, we defined nine epochs (see^41^):

(1) *Baseline*: from 750 to 250 ms before the onset of the instructing cue; (2) *Pre-cue*: 250 ms preceding the onset of the instructing cue; (3) *Cue*: 250 ms following the onset of the instructing cue); (4) *Pre-presentation*: 500 ms preceding the opening of the box door; (5) *Presentation*: 500 ms following door opening (object presentation); (6) *Set*: 250 ms before the offset of the instructing cue; (7) *Go/NoGo*, from the offset of the instructing cue to the release of the hand starting position (Action condition) or 250 ms following the offset of the instructing cue (Inaction condition); (8) *Grasping*-*Pulling/Object fixation*: from 250 ms before to 250 ms after the Pulling onset (Action condition) or a time period ranging from 250 to 500 ms after the offset of the instructing cue (Inaction condition); (9) Reward: 500 ms following reward delivery.

Single-neuron responses were statistically evaluated by means of a 9X2 ANOVA for repeated measures (Factors: Epoch, Condition, *p* < 0.01) followed by Newman–Keuls post hoc tests. In this study, neurons were considered as visually responsive when there was a significant main effect Epoch (*p* < 0.01), and the following post-hoc test showed a significant difference between the presentation and the baseline epochs.

The comparison between the Visual and the Visuo-Motor tasks was performed on those neurons considered as visually responsive in at least one task (according to the criteria defined in the previous sections) and whose neural activity was recorded in 90 successful trials, 10 for each stimulus (Cube, Cylinder and Sphere) in each condition (Visual, Action and Inaction). Single-neuron responses were statistically evaluated by means of a 3X3 ANOVA for repeated measures (Factors: Conditions, Stimuli, < 0.01) followed by Newman-Keuls post hoc tests.

Since the Visual and Visuo-Motor tasks were acquired in different blocks, to ensure that baseline activity was not changed across tasks, we compared the activity of each neuron during the period preceding the stimuli presentation of both tasks. To this aim we conducted a t-test between the baseline epochs of the Visual task and the pre-presentation epoch of the Inaction condition of the Visuo-Motor task. Note that in these two epochs the monkey had to keep fixation on the same red fixation point and was not required to program any movement. We discarded all neurons showing a significant difference between these two epochs.

### Computation of depth of tuning, selectivity and category indexes

To quantify the degree and depth of selectivity of the neurons for the images used in the Visual task, we calculated two indexes: the depth of tuning index (d_i_), measuring the difference between the maximal and the minimal response normalized to the cell’s maximal response, and the selectivity index (s_i_), quantifying the extent to which activity in all non-preferred stimuli deviates from the maximal activity for the preferred stimulus. Each index was computed for each neuron across the twelve stimuli employed. These indexes, previously employed for assessing the neuronal directional tuning^45^, are defined as follow:

$$d_{i} = \frac{{i_{max} - i_{min} }}{{i_{max} }}$$

$$s_{i} = \frac{{k - \left( {\frac{{\mathop \sum \nolimits_{n = 1,k} i_{n} }}{{i_{max} }}} \right)}}{k - 1}$$

where *k* is the number of stimuli; and *i*_*min*_ and *i*_*max*_ are, respectively, the minimum and maximum responses of the *i*th neuron across the twelve different stimuli and are calculated based on the peak of discharge observed in the stimulus epoch.

### Population analyses

In order to characterize the time course and the discharge rate of different neuronal populations, the neuronal activity of each population was aligned with the beginning of Stimulus epoch (Visual task) or the actual objects appearance (Visuo-Motor task). The population activity was computed as follows. The mean single neuron activity over trials, in terms of firing rate, was calculated for each 20 ms bin in the different conditions. The average Baseline (Visual task) or Pre-presentation epoch (Visuo-Motor task) activity was then subtracted from the mean single neuron activity over trials for each bin. Each neuron contributed one entry to each data set. In the Visual task, each bin represents the average activity recorded in the 12 stimuli of the number of neurons included in the population. Due to mechanical timing of door opening in the Visuo-Motor task, the object became visible 38 ms after door opening. Since population analysis was calculated in bins of 20 ms, we aligned population activity in the Action and Inaction conditions two bins (40 ms) after door opening.

In order to assess whether the population activity recorded in the Stimulus epoch of the Visual task statistically differed among the 12 presented stimuli, we conducted a One-way ANOVA, Tukey–Kramer criterion, *p* < 0.01 (Matlab functions: anova1, multcompare). Similarly, we compared the population activity recorded in the Stimulus epoch of the Visual task and in the Presentation epoch of the Visuo-Motor task by means of a One-way ANOVA, Tukey–Kramer criterion, *p* < 0.01 (Matlab functions: anova1, multcompare).

### Anatomical reconstruction of the neuronal properties

The recording region was reconstructed based on the location (in stereotaxic coordinates) of the penetrations on the MRI scans of the brain of both investigated monkeys, as described in^41,43^. Penetration depth, as reported by the protocol, was matched with its location with respect to the sulci.

## Results

This study had two main aims: the first was to describe and map the responses of VLPF neurons to the observation of a set of visual stimuli, with the only requirement for the monkey to simply fixate the image, the second was to assess whether prefrontal neurons respond differently to real objects or to their pictures and activate differently when these objects are passively observed or are target of an action. Accordingly, we will first present the results obtained in the Visual task, and subsequently those derived from the comparison of neural responses recorded with the Visual and the Visuo-Motor tasks.

We recorded 1607 neurons from VLPF during performance of the Visual task.

**Figure 2:**
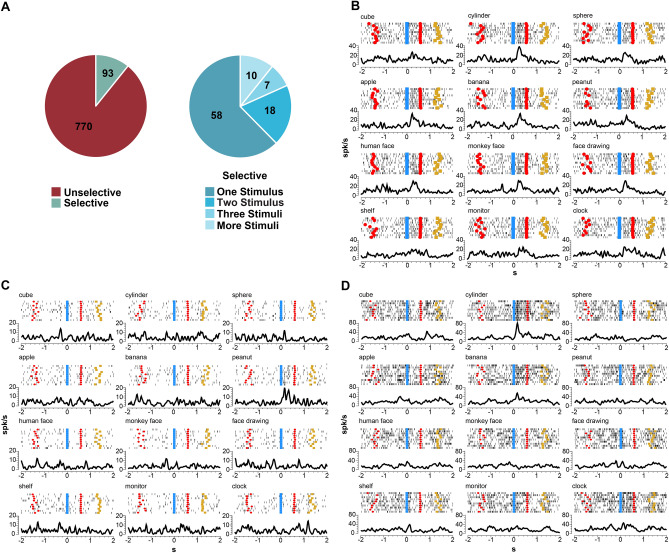
Selective and Unselective neurons. (**A**) Pie plots showing the proportion of Visual Neurons classified as Selective or Unselective (left) and the number of neurons selective for 1, 2, 3 or more stimuli (right). (**B**) Example of neuron responding to all the presented stimuli. (**C**) Neuron responding exclusively during the observation of a Peanut. (**D**) Neuron responding to the observation of Cylinder and Banana. The neuronal activity represented by rasters and histograms is aligned on the beginning of stimulus presentation (0). In each raster, cyan squares indicate the beginning of stimulus; red circles indicate the switching on of the red laser light; yellow squares indicate reward delivery. Abscissae: time (s); Ordinates: firing rate (spikes/s).

The 2 × 12 ANOVA for repeated measures (Factors: Epochs, Stimuli, *p* < 0.01, see Methods) reveals, that, of these neurons, 863 were visually responsive. The majority was active during the observation of all stimuli, while 93 neurons (10.8% of visually responsive neurons, Fig. 2A) had an interaction effect, showing some type of preference for one or more stimuli (selective neurons, see Methods). Figure 2A shows also the number of neurons selective for 1, 2, 3 or more stimuli. It is clear that most of them (n = 58, 62%) responds only to one stimulus.

Figure 2B–D depicts examples of three visually responsive neurons. Figure 2B shows the discharge of an unselective neuron, responding equally well to all the presented stimuli (see “Methods” section). In Fig. 2C, a neuron responding only to the presentation of a peanut is shown. With respect to image onset, the discharge begins at about 100 ms, peaks at about 200 ms, and ends before image offset. Figure 2D shows the response of a neuron selective for two stimuli (Cylinder and Banana). Note that there is no significant difference between them. The discharge for both stimuli begins and peaks shortly after image onset, and declines just after image offset. Interestingly, although the two stimuli belong to different categories, they share common features, namely shape and orientation.

Table 1 shows the number of neurons grouped on the basis of their best response to the presented stimuli. One can appreciate that, although all stimuli are coded by the selective neurons, some stimuli are coded by a higher number of neurons. However, it is also clear that the best neural responses fall almost equally within the different semantic categories set a priori in our experiment of stimuli. (χ^2^ (3) = 6.4659, *p*-value = 0.09102). In addition, note that the neuron selectivity for specific stimuli does not seems to correlate with the difference in peak-firing rate or in the timing of peak discharge (Supplementary Fig. 1).

**Table 1:** Number of selective neurons grouped on the basis of their best response to each stimulus.

Stimulus	n. neurons	Stimulus	n. neurons	Stimulus	n. neurons	Stimulus	n. neurons
Cube	2	Apple	6	Human face	7	Shelf	7
Cylinder	13	Banana	13	Monkey face	7	Clock	7
Sphere	5	Peanut	12	Face drawing	3	Monitor	11
Solids	20	Fruits	31	Faces	17	Furniture	25

The best response is the highest among the coded stimuli.

Concerning the possibility of neurons to categorize, among those responding to more than one stimulus, none discharge during observation of the three stimuli belonging to one pre-set semantic category and not to those belonging to the other three categories; 8 neurons respond to two of the three stimuli belonging to only one semantic category.

**Figure 3:**
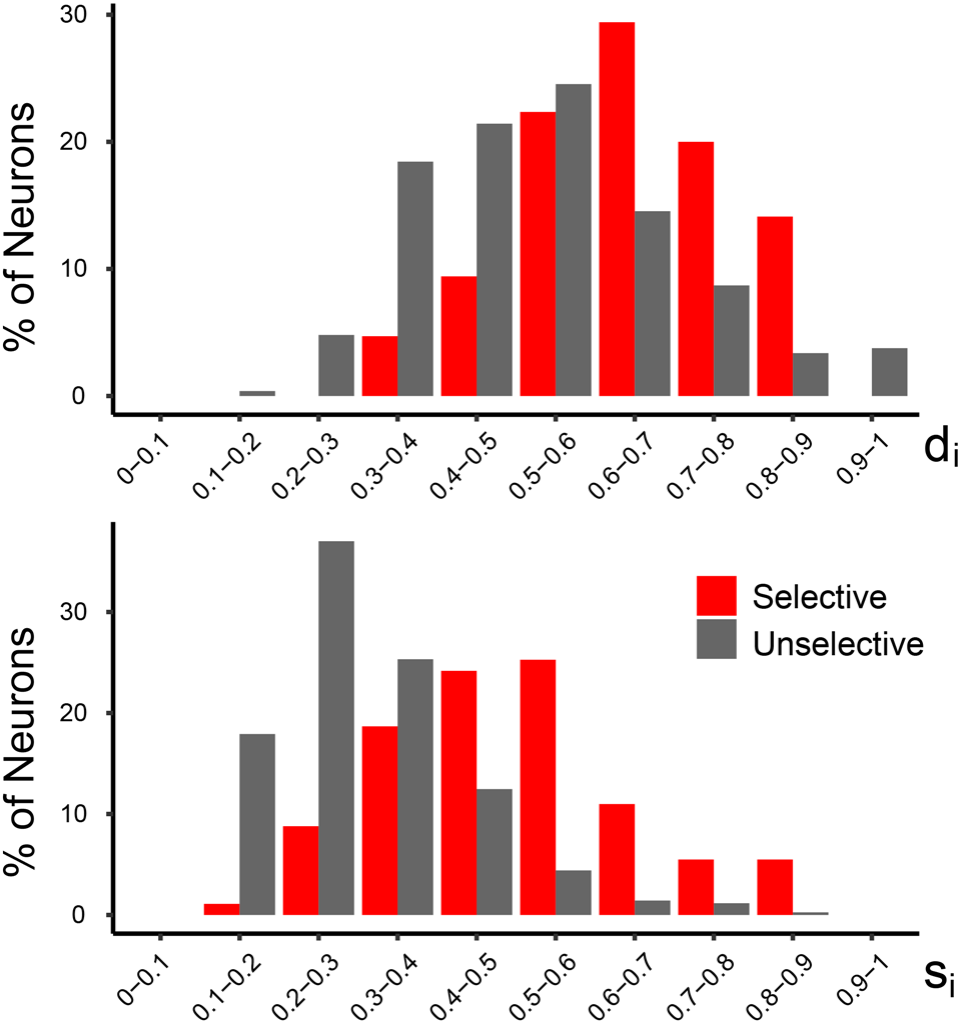
Histograms showing the distribution in percentage of selective (red) and unselective (gray) neurons based on d_i_ (**A**) or s_i_ (**B**). The value of each bin in the abscissa corresponds to a range of 0.1 for each index.

In order to quantify the stimulus selectivity of the neurons, we calculated two indexes (see Methods). The depth of tuning index (d_i_) indicates how sharply each neuron codes the best stimulus with respect to the worst, and the selectivity index (s_i_) quantifies the extent to which activity in all non-preferred stimuli deviates from the maximal activity for the preferred stimulus, indicating how much each neuron is tuned to one stimulus. Figure 3 shows the results of the calculation of depth of tuning (d_i_) and selectivity (s_i_) indexes for selective and unselective neurons. The average d_i_ is higher (0.72 ± 0.16) for selective with respect to unselective neurons (0.53 ± 0.17), indicating a larger difference in discharge between the best and worst stimuli in the selective neurons. As far as s_i_ is concerned, its average is clearly higher for selective neurons (0.49 ± 0.15 vs 0.31 ± 0.12).

### Population analyses

**Figure 4:**
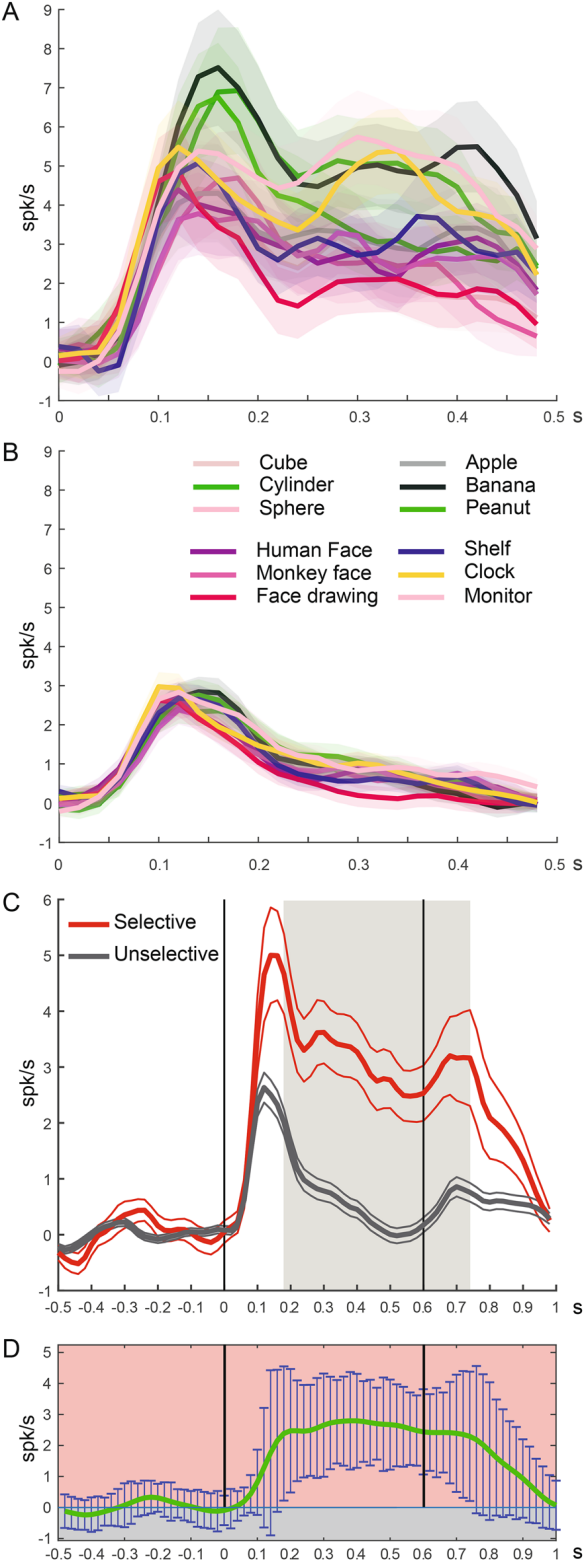
Temporal profile of the net mean activity of the populations of selective (**A**) and unselective (**B**) neurons during the observation of visual stimuli. The different colored lines indicate the populations net mean activity for each stimulus, the shaded colored contours represent the standard errors. The activity is aligned (0) with the stimulus onset. (**C**) Temporal profile of the net mean activity of the populations of selective (red) and unselective (gray) neurons in the visual task, averaged among the 12 stimuli. The thick lines indicate the populations average activity, the thin lines represent the standard errors. The shaded area represents the temporal span in which the two activities differ significantly. (**D**) Temporal profile of the net differential activity calculated as subtraction between the average activities of Selective and Unselective populations. Error bars indicate three times the standard error for each 20 ms bin. The vertical lines indicate the onset and offset of stimulus presentation. Abscissae: time (s); Ordinates: firing rate (spikes/s).

Population analyses were conducted on selective and unselective neurons. Figure 4A shows that the average discharge intensity, among selective neurons, is higher for those stimuli that are encoded by a higher number of neurons (One way ANOVA, *p* < 0.01). On the contrary, statistical analysis did not show any difference in discharge in unselective neurons (Fig. 4B, One way ANOVA: n.s.).

Figure 4C shows the comparison in the time course of the discharge, averaged among all stimuli, between the two populations. The beginning of the response is the same for both curves, while the maximal raising slope (calculated as the maximum of the derivative) and the peak of activity is slightly earlier (20 ms) for the population of unselective neurons (80 vs100 and 120 vs 140 ms after stimulus onset, respectively). Then, after an initial rapid decrease in the discharge similar in both populations, the two profiles have a different time course. While the population of unselective neurons returns to baseline level before stimulus offset, that of selective neurons presents a sustained discharge falling to baseline level only during the second fixation epoch. Note that both populations present a second minor peak after stimulus offset, in coincidence with the second appearance of the fixation point. The statistical comparison between the two population discharges shows that they start differing 180 ms after stimulus onset, just after peak of discharge. Differential discharge ends 140 ms after stimulus offset (Fig. 4D).

### Localization of selective neurons

**Figure 5:**
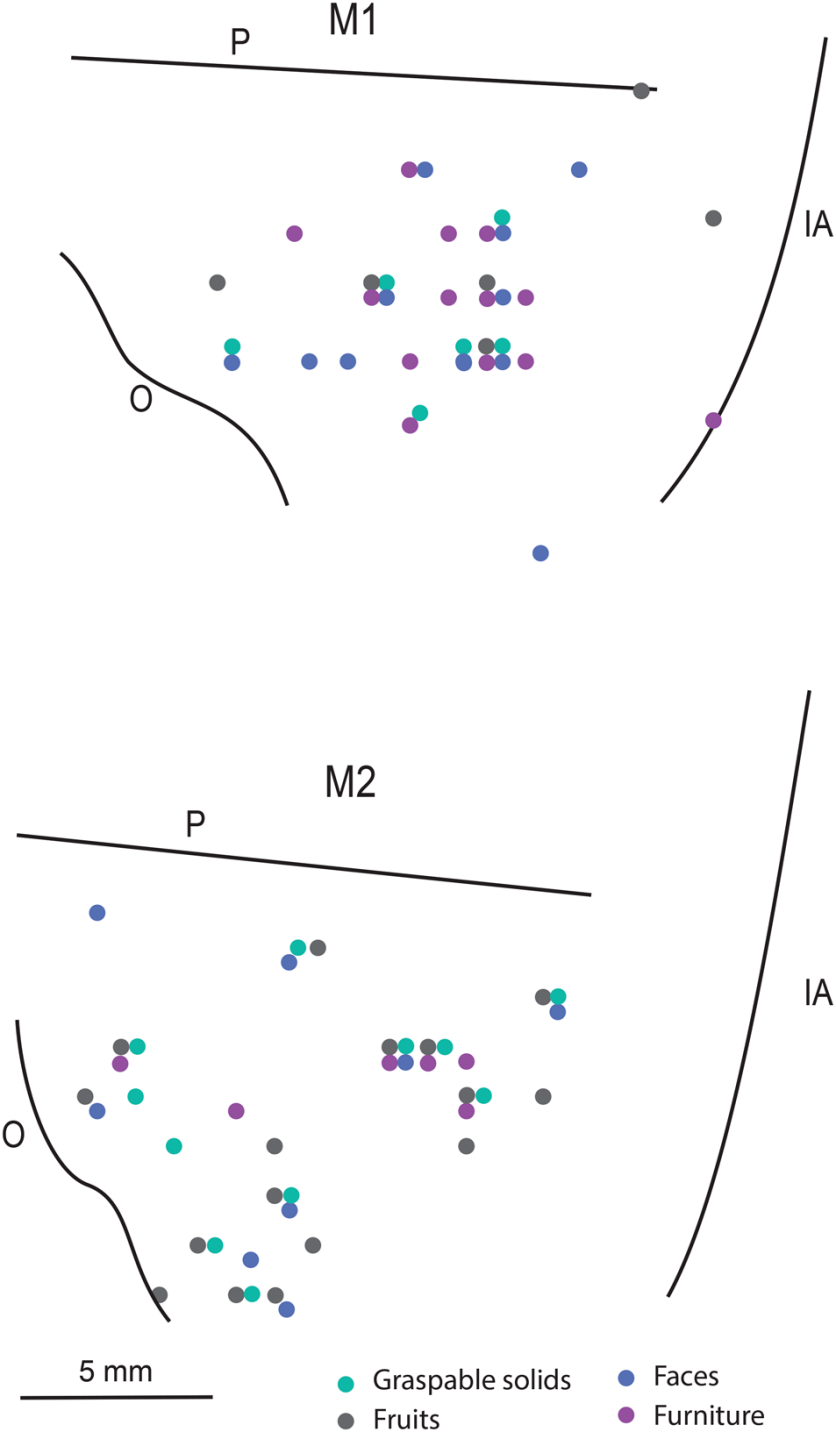
Distribution of penetrations containing neurons with selective discharge for visual stimuli belonging to different categories in the recorded region of the two monkeys (M1 and M2). IA, inferior arcuate sulcus; O, orbital reflection; P, principal sulcus.

Figure 5 depicts the distribution of neurons showing selective discharge for visual stimuli belonging to different categories in the two monkeys. Note that in both monkeys the selective neurons are localized over a wide region likely corresponding to areas 46, 12 and 45. In monkey 2 there is an additional cluster of neurons close to oculomotor areas. No evident segregation is apparent among the various classes of stimuli.

### Comparison between responses to objects observation in the visual and Visuo-Motor tasks

#### Single neurons responses

**Figure 6:**
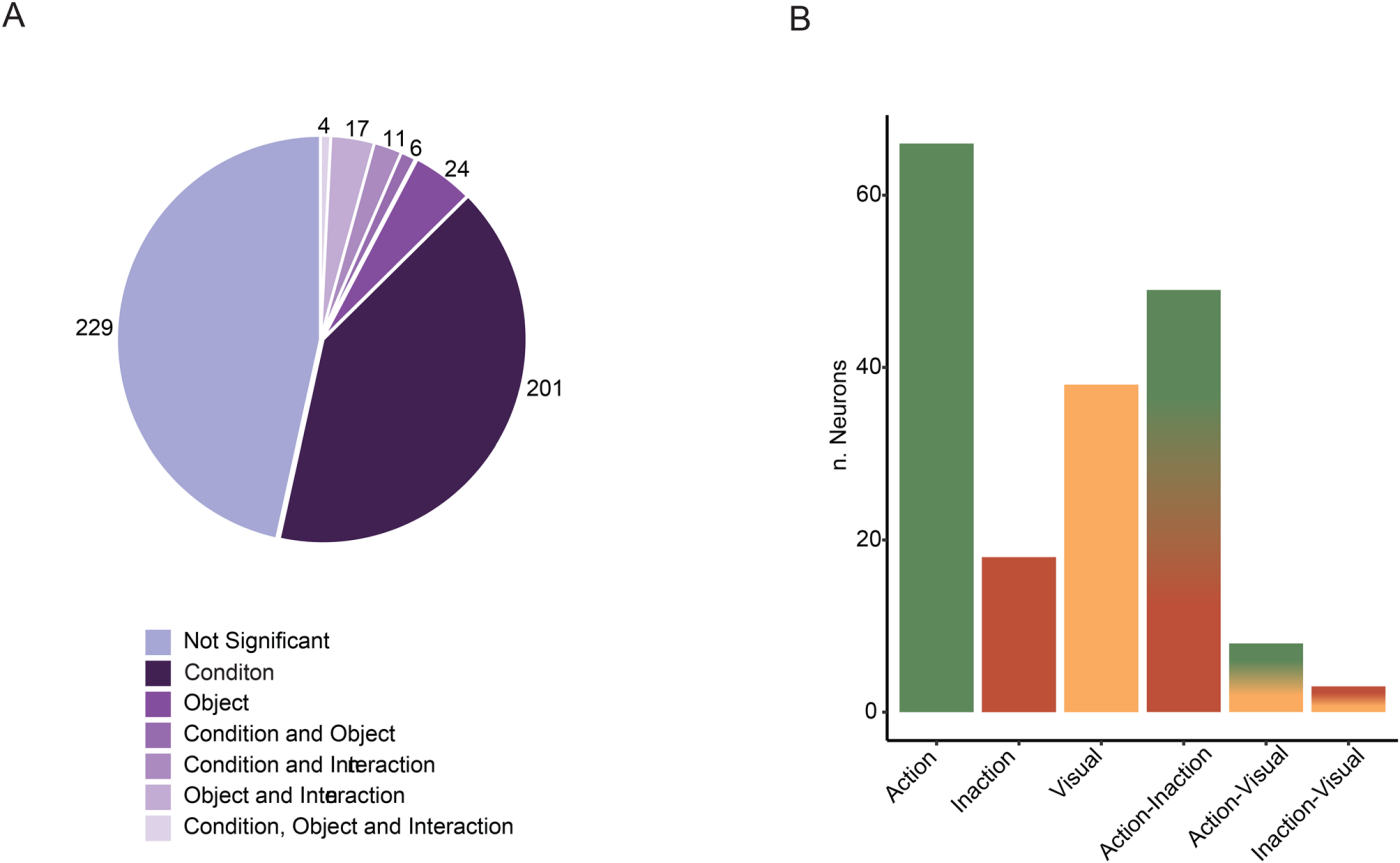
(**A**) Pie plot showing the distribution of neurons according to the results of statistical analysis (3 × 3 ANOVA for repeated measures; factors: Condition and Object). (**B**) Histograms representing the distribution of neurons showing differential activity during object observation in the Action, Inaction and Visual conditions. Single-coloured histograms indicate neurons responding better in one condition than in the other two; double coloured histograms indicate neurons responding equally well in two conditions and better than the remaining one (see text for the detailed description of each category).

The responses of 492 neurons tested in both Visual and Visuo-Motor tasks were analyzed using a 3X3 ANOVA for repeated measures (factors: condition and object, *p* < 0.01) in order to verify the effect on neural discharge of the context in which the stimuli were observed and of the type of object. Figure 6A shows the distribution of neurons according to the results of this statistic analysis. Briefly, about half of the analyzed neurons (n = 242) had a significant condition effect, 11% (n = 56) had a significant object effect and only a minority of neurons (4%, n = 21) had an interaction effect; 229 neurons (46.5%) had no significant effect.

**Figure 7:**
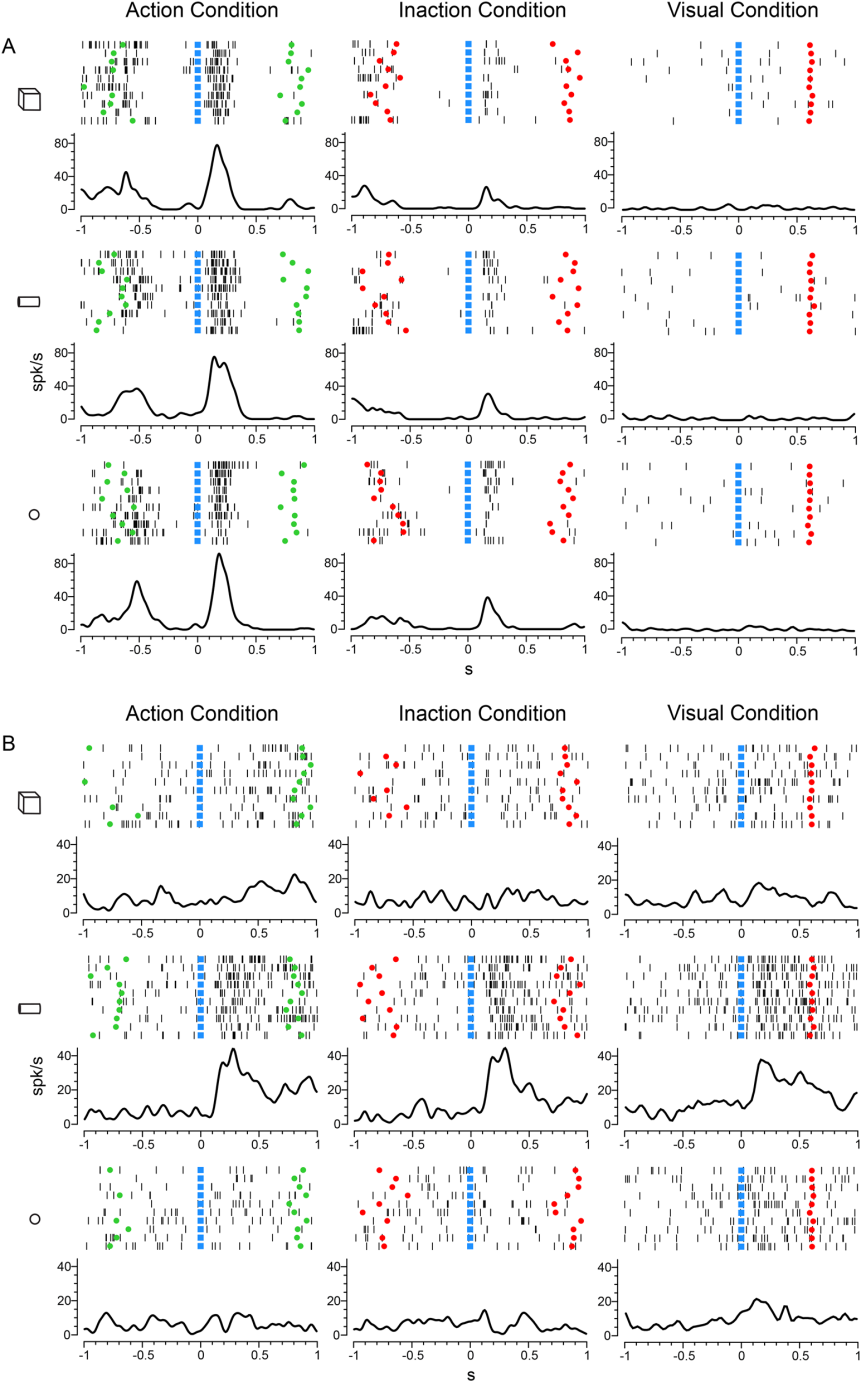
(**A**) Example of VLPF neuron discharging strongest during object presentation in the Action condition. (**B**) Example of VLPF neuron responding selectively to the observation of the cylinder in all conditions. For both neurons the activity is aligned on the beginning of stimulus presentation. Green circles indicate the switching on of the green laser light. Other conventions as in Fig. 2.

The majority of neurons with main effect condition (n = 182, 75%) showed a significant difference between one condition and the other two (Fig. 6B). Among them, we recognized three first categories based on the condition better coded than the other two. The most represented category is that formed by neurons discharging during object presentation in the Action condition (n = 66). An example of neuron belonging to this category is shown in Fig. 7A. The neuron strongly activates during object observation in the Action condition, has a much weaker activation in the Inaction condition, and does not respond at all in the Visual condition. The neuron does not show any object selectivity. Among the neurons preferring the Action condition, 4 showed object preference and 7 resulted also active during movement execution (according to the criteria adopted by Simone et al.^41^). A second category is formed by neurons responding best to object presentation in the purely Visual condition (n = 38). A third category is formed by neurons responding best to object presentation in the Inaction condition (n = 18).

Then, we could recognize three further categories formed by neurons coding two conditions better than the remaining one. The first is formed by neurons that respond better in the Visuo-Motor task than in the Visual task (n = 49). The second contains neurons that respond in the Action and purely Visual condition better than in the Inaction condition (n = 8). The third is formed by neurons responding in the Inaction and Visual condition better than in the Action condition (n = 3).

The remaining 60 neuron (25%) showed a significant difference only between two conditions, the other comparisons being not significant.

Neurons showing condition effect can be classified in a different way, based on object dimensionality (2D vs 3D)/task complexity (Visuo-Motor vs Visual). Thus, we calculated the number of neurons falling within this macro-category. We found that 85 out of 242 neurons (35%) belong to it. On the contrary, 105 neurons (43%) show a significant difference between Action and Inaction conditions, thus clearly do not code the considered aspects. The remaining 52 neurons do not fit this categorization.

Neurons showing an object and/or an interaction effect in the 3X3 ANOVA have been categorized based on their best response. This categorization shows that 39 neurons have a highest discharge for the cylinder, 11 for the cube and 10 for the sphere. Note that four neurons had the same best discharge for two objects. Figure 7B shows an example of a neuron selective for the cylinder in all conditions. The response for the other two objects is much weaker or absent.

#### Population response

**Figure 8:**
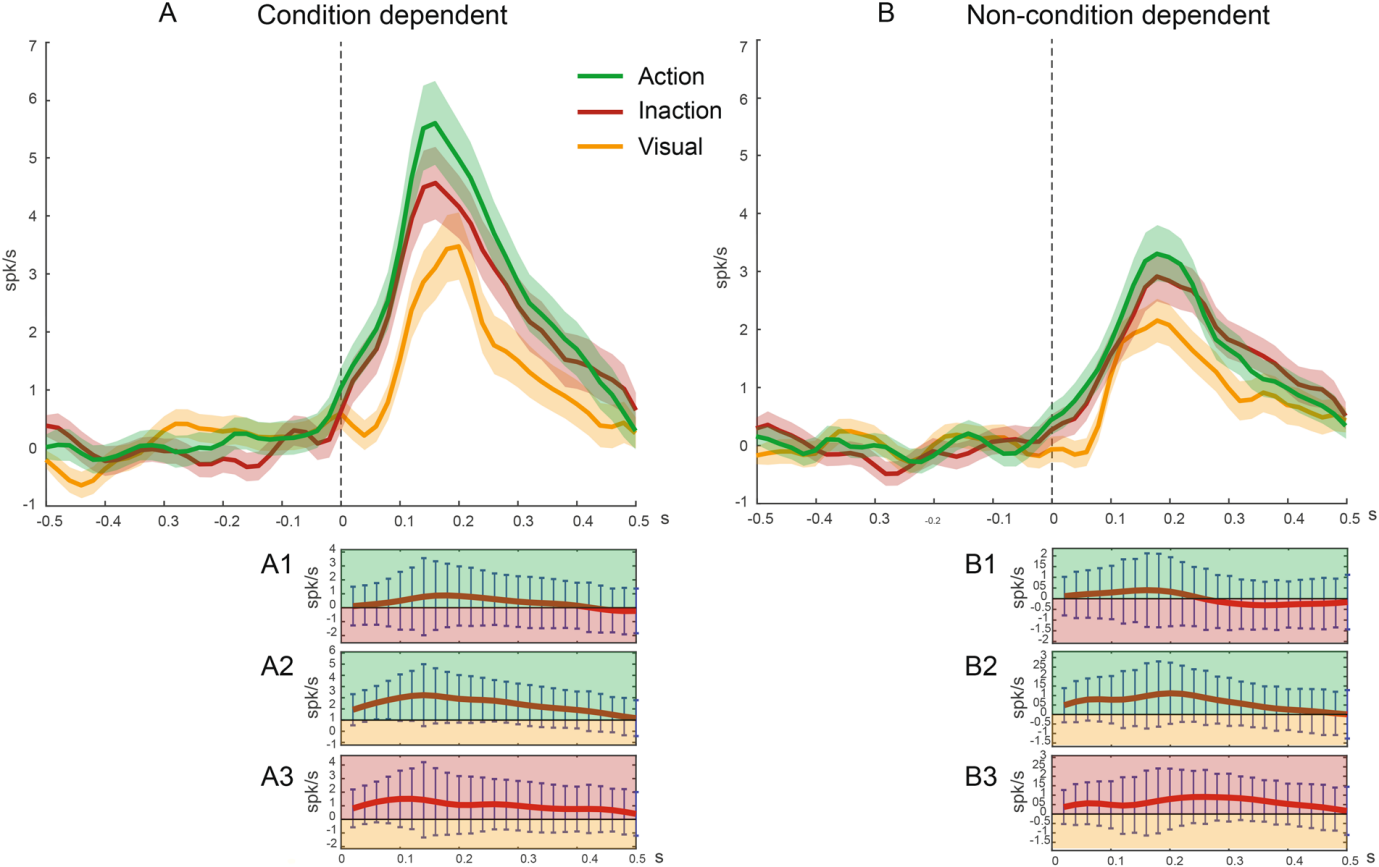
Temporal profile of the net mean activity of the populations of Condition dependent (**A**) and Non condition-dependent (**B**) neurons during the observation of visual stimuli in the Action, Inaction and Visual conditions. The colored lines indicate the population net mean activity for each condition, the colored shaded contours their standard errors. The activity is aligned (0) with the stimulus onset. Temporal profiles of the net differential activity calculated as Action minus Inaction (**A1**, **B1**); Action minus Visual (**A2, B2**); Inaction minus Visual (**A3, B3**) of the condition dependent and non-condition dependent populations. Other conventions as in Fig. 4.

Figure 8 shows the time course of the population responses of the neurons recorded in the three conditions. The population of neurons with a significant condition effect (Fig. 8A) shows the highest discharge for objects observation during the Action condition, an intermediate discharge for the Inaction condition, and the weakest response for the Visual condition. The statistical analysis reveals a significant difference among conditions and that the Action condition has a stronger response than the Visual one (one way ANOVA, Tukey–Kramer criterion, *p* < 0.01). The plot of the time course of the differential activity between pairs of conditions confirms a significant difference only between the Action and the Visual condition in two bins preceding the peak of the activity in the Action condition (Fig. 8 A2).

The population of neurons without a condition effect (Fig. 8B) shows a similar trend, although the discharge in the Action and Inaction condition is quite similar. The one way ANOVA (Tukey–Kramer criterion, *p* < 0.01) does not reveal any significant difference among conditions. The plot of the time course of the differential activity between pairs of conditions does not reveal any significant difference.

Looking at the time course of the two populations, it is worth to make two further considerations. First, the peak of activity is higher in the condition dependent population; second, while in the non-condition dependent population the timing of peak in the three conditions is perfectly aligned (160 ms after object appearance), in the condition dependent population, in Action and Inaction condition, the peak occurs before that of the Visual condition (140 vs 180 ms after object appearance).

### Localization of neurons selective for the action, inaction and visual conditions

**Figure 9:**
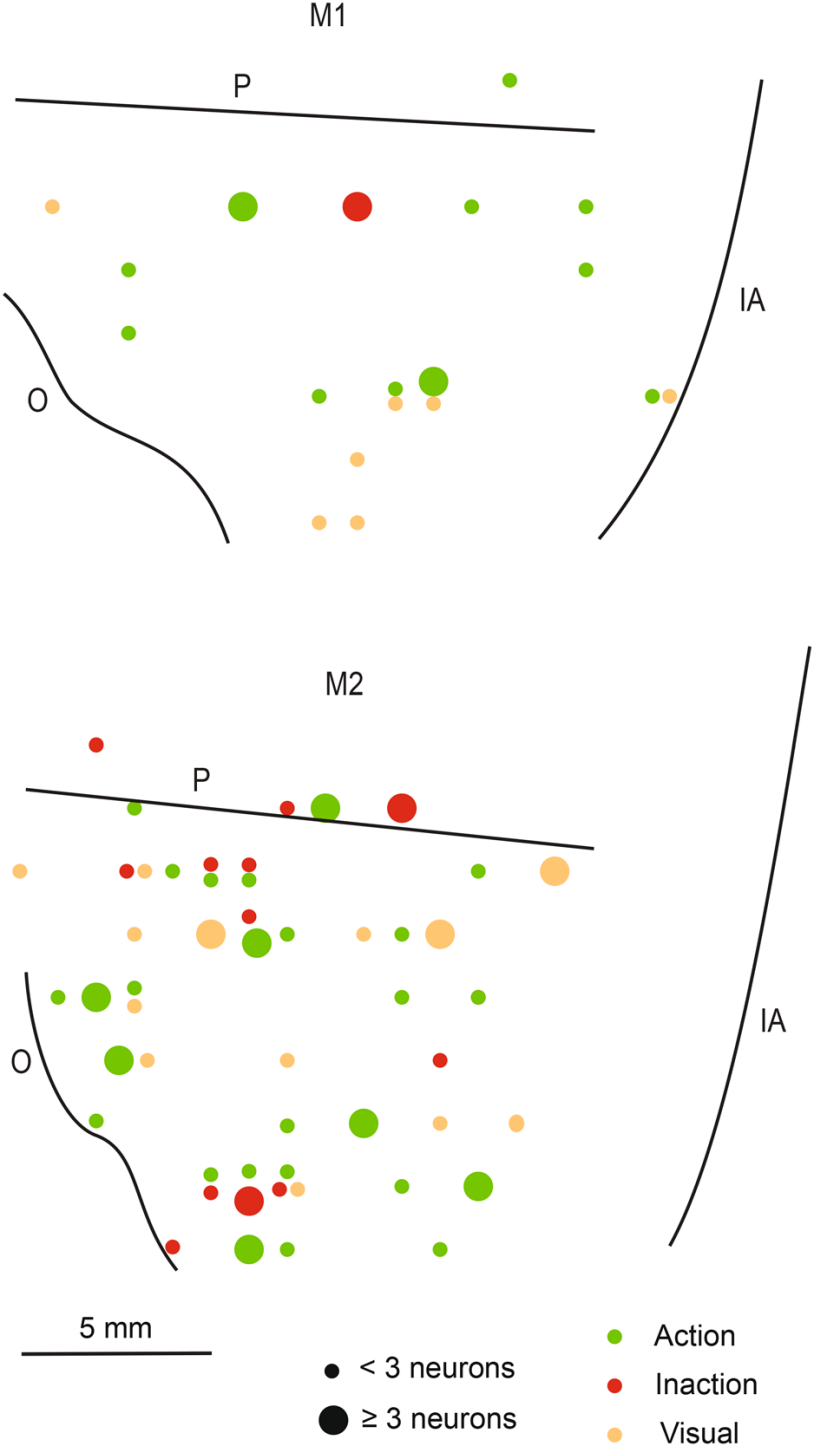
Distribution of neurons showing selectivity for one of the conditions in the recorded region of the two monkeys (M1 and M2). Different size of the circles indicate different numerosity of neurons. IA, inferior arcuate sulcus; O, orbital reflection; P, principal sulcus.

Figure 9 depicts the distribution of neurons showing selectivity for one of the conditions in the two monkeys. The two maps are different in terms of amount of neurons showing selectivity. Even the map of the monkey showing the largest number of neurons (M2) does not show any clear segregation between the three types of selective neurons. Indeed, they are localized over a wide region covering areas 46, 12 and 45.

## Discussion

The results of the first part of the present study, aimed to describe the VLPF neurons responses to different types of passively presented visual stimuli, show that: (a) about half of the recorded neurons respond to visual stimuli; about 10% of them also show stimulus selectivity, the majority being selective for only one stimulus; (b) the main categories of the used stimuli are equally represented; (c) the time course of the discharge of the populations of both selective and unselective neurons presents a peak at about 130 ms after stimulus onset, but only selective neurons show a prolonged discharge for the whole duration of the stimulation; (d) visually responding neurons are widely distributed within the recorded region, covering areas 45A, 46v, 12, sparing only FEF.

The results of the second part of the study, concerning the comparison between neuronal responses to objects observation in the passive observation task (Visual condition) and in the visuo-motor task (Action and Inaction conditions) show that: (a) about half of the tested neurons show a differential discharge between conditions; (b) the most represented category is that of neurons responding best during the Action condition; (c) the time course of the population response shows the highest activation in the Action condition, an intermediate activation in the Inaction condition and the lowest activation in the Visual condition; (d) there is no specific anatomical segregation within the recorded region for neurons responding best to a single condition.

### Visual selectivity in VLPF neurons

The presence of selective neurons in VLPF is in good agreement with the literature on the passive response of prefrontal cortex to visual stimuli, reporting that the percentage of neurons showing visual specificity is quite limited. It is noteworthy that in most cases neurons were tested with a restricted number of stimuli^13,21,46^. On the other hand, it has also been reported that in VLPF there are neurons with very specific responses to complex stimuli such as faces either static, or dynamic in combination with vocalizations^38,39,46–48^. In our study, we employed a set of stimuli larger than those previously employed in studies on prefrontal cortex. This allowed us to show that most of the employed stimuli are specifically coded by VLPF neurons. Very likely, by using an even larger set of stimuli, a higher number of selective neurons could have emerged.

The fact that in our study neurons responding to faces were not so represented as in previous works, could partly depend on the wider region we recorded. In addition, previous studies showing responses to faces actually used only faces as stimulus^38^, so we do not know if these neurons could have responded to other visual stimuli, too.

Some studies demonstrated the capacity of VLPF neurons of categorizing visual stimuli ^1^. This appears in contrast with our findings, showing that most selective neurons respond to only one stimulus, and that the stimuli coded by neurons responding to more than one stimulus belong to different categories. A possible explanation of the lack of categorical generalization is that we concentrated on specific semantic categories, excluding other possible stimuli. Another possibility could be related to the task we employed, that did not require to perform any active operation referred to the observed stimuli, as done in other studies.

An interesting finding emerging from our study is that in VLPF it is possible to elicit neuronal responses using passive stimulation. This probably reveals the contribution of inferotemporal and posterior parietal input to prefrontal cortex. This input represents the first step in the intrinsic prefrontal processing. Indeed, it has been shown that when presentation of visual stimuli is compared between a passive observation task and a task in which the stimulus is an instruction for a subsequent response, there are neurons whose visual response does not change and others that do show a response only in the active condition^2,20,49,50^.

The visually selective neurons found in the present study are evenly distributed in the recorded region, thus encompassing areas 45A, 46v and 12. No segregation was detected for either specific stimuli or categories. This finding is quite in agreement with the literature in which the same cortical sector was explored^6,39,51^. Thus, it looks as if the incoming temporal and parietal visual input reaches prefrontal cortex without a specific organizational pattern, such as, for example, similar shapes, categories or affordances. Note, however, that the possibility that some type of organization of visual responses in VLPF does exist comes from the demonstration of the presence, in ventral and orbital prefrontal cortex, of three patches activated by face observation^22^, in line with the patchy representation of faces in inferotemporal cortex.

Population analysis clearly reveals that both selective and unselective neurons have a first peak of response with a similar timing. This could be partly attributed to an initial attention-related response or to an activity related to a specific, crucial, phase of task unfolding^2,3,6,52–55^. These explanations could be completely valid for the second peak of discharge occurring after stimulus offset when the fixation point reappears. On the contrary, in the case of the first peak, the population of selective neurons has twice the discharge of that of unselective neurons. Thus, if the peak were related only to unspecific factors, or phases, one would expect exactly the same discharge intensity in both populations. On the contrary, it seems that selectivity is the main factor capable of explaining this difference. Furthermore, only in the population of selective neurons the response remains sustained for the whole presentation period.

Regarding the role of the sustained discharge, we can exclude that it can be attributed to memory, since in this period the stimulus is always visible. In addition, it cannot be due to some sort of preparation, since the monkeys have just to passively observe the visual stimuli. Another possible explanation for this discharge is that it contains some sort of expectation of the next phase of the task (second fixation), since visual presentation lasts a fixed period of time. This seems unlikely because if this were the case the discharge should increase before stimulus offset. The interpretation we favor is that the sustained discharge represents a top-down activation of the temporal/parietal neurons that provided the visual specific information. This would serve to maintain attention on the target^2,56–59^.

### VLPF neurons responding to objects observation play a role in planning behavior

The second part of the study was aimed to verify whether the same types of objects presented in the Visual or Visuo-Motor task could elicit the same or a different response in the VLPF recorded neurons.

This comparison shows that half of the tested neurons did show a different discharge among conditions. In order to better understand the type of coding of these neurons, we can first consider neurons showing specificity for only one condition. The majority of them has the best activation for the Action condition, followed by those preferring the Visual condition, while the minority activates best for the Inaction condition. The large prevalence of neurons preferring the Action condition suggests that the response occurring during visual presentation is clearly related to the action the monkey is going to perform on the object. This does not mean that the neuron must show a discharge during action execution (only 7 neurons show movement related discharge), but rather that the neuron is involved in a neuronal chain whose activation leads to action-goal achievement. Note that these neurons do not have a role in visuo-motor transformation, as it is also confirmed by the very low number of them having an object preference in the Action condition (n = 4). The absence of this role was already demonstrated in a previous work on movement related neurons of this same region using the same task^41^. A similar explanation can apply to the role of neurons preferring the Inaction condition, although in this case the effect is the opposite. In fact, the response to the object has the meaning of action withholding. Thus, very likely these neurons participate to the inhibition of neuronal chains activated by this same object. This role is in complete agreement with the well-established function of the prefrontal cortex to inhibit unwanted actions (see^2,3,32^). Concerning neurons preferring the Visual condition, the most likely explanation is that their discharge is related to the 2D features of the stimulus as provided by the inferotemporal cortex (see^60^). These neurons could have the role of emphasizing the inferotemporal-dependent object recognition for its possible use in abstract tasks (e.g. categorization, stimulus-reaction association).

A certain number of neurons respond equally well to two conditions and higher than the remaining one. Two categories are worth to be discussed. The first, larger category is made by neurons responding well to Action and Inaction conditions, their activation being weaker or absent in the Visual condition. The type of coding of these neurons could be related either to the 3D versus 2D features of the object or to task complexity (Visual vs Visuo-motor). The second category is formed by neurons responding stronger to the Action and the Visual condition, with a weak or absent discharge during Inaction condition. The type of coding of these neurons would be related to a situation in which action inhibition is not requested.

Overall, the various types of preference shown by single VLPF neurons indicate that several factors can influence the response to object observation. This is very likely related to the fact that this region on the one hand reflects visual inputs coming from high order visual cortex, on the other the possible meaning of these inputs when exploited for specific behaviors.

The population analysis provides a wider view on the function of the recorded region. Indeed, object observation in the Action condition produces the highest population response, followed by that in the Inaction condition. Object observation in the Visual condition elicits the weakest discharge. This suggests that the highest the behavioral relevance of the object, the strongest the prefrontal neuron response. This is in agreement with the literature showing higher neuronal responses when a visual abstract cue becomes associated to a specific behavioral response^61,62^, see^49^. Our data show that this concept applies also when the visual stimulus is a graspable object.

## Conclusion

The results of the visual presentation of several types of stimuli belonging to different categories confirm the strong impact of inferotemporal cortex input on VLPF, in line with the data from neuroanatomical studies^63,64^. A wide literature suggested that VLPF exploits this inferotemporal input for categorization and task instruction see^2,3,7^. The fact that visually responding neurons are present not only in the areas typically target of inferotemporal cortex such as areas 12r and 45A, but also in 46v, is very likely due to the fact that, in principle, part of used visual stimuli can be the target of goal directed actions. Indeed, 46v and 12r are strongly connected with inferior parietal areas such as AIP, PFG and SII, all involved in hand related actions. This is confirmed by the data of our comparison between objects presentation in either the passive Visual task or in the active Visuo-Motor task.

The differential response of half of tested neurons to Visual, Action and Inaction conditions allows to speculate on their possible activation in a network involved in acting on objects.

The connections between lateral prefrontal cortex and both posterior parietal and inferotemporal cortices can provide the former with both a pragmatic and a pictorial description of the object, so that VLPF, based on different types of instructions can employ this information to guide behavior.

Our previous data showed that in this prefrontal sector there are neurons discharging both during object presentation and grasping^41^. In the neuronal set tested in the present study, many neurons responding better to objects in the Action condition have no motor response. However, their differential discharge suggests that they can produce an output necessary to guide an object related action. Their role in this output depends on the neurons to which they are connected, either other neurons of VLPF, parietal neurons or premotor neurons. Another possible use of this output is a feedback projection to the temporal neurons involved in coding the properties of these objects, keeping active their perception during action selection.

## Supplementary Information

Supplementary Information 1.
