# Supplementary material for: Visual response of ventrolateral prefrontal neurons and their behavior-related modulation
Source: Sci Rep. 2021 May 12;11:10118. doi: 10.1038/s41598-021-89500-0 (PMC8115110; doi:10.1038/s41598-021-89500-0)
Supplement: Supplementary file 1 — Supplementary Information 1. [file 41598_2021_89500_MOESM1_ESM.docx]

**Supplementary Figure 1**


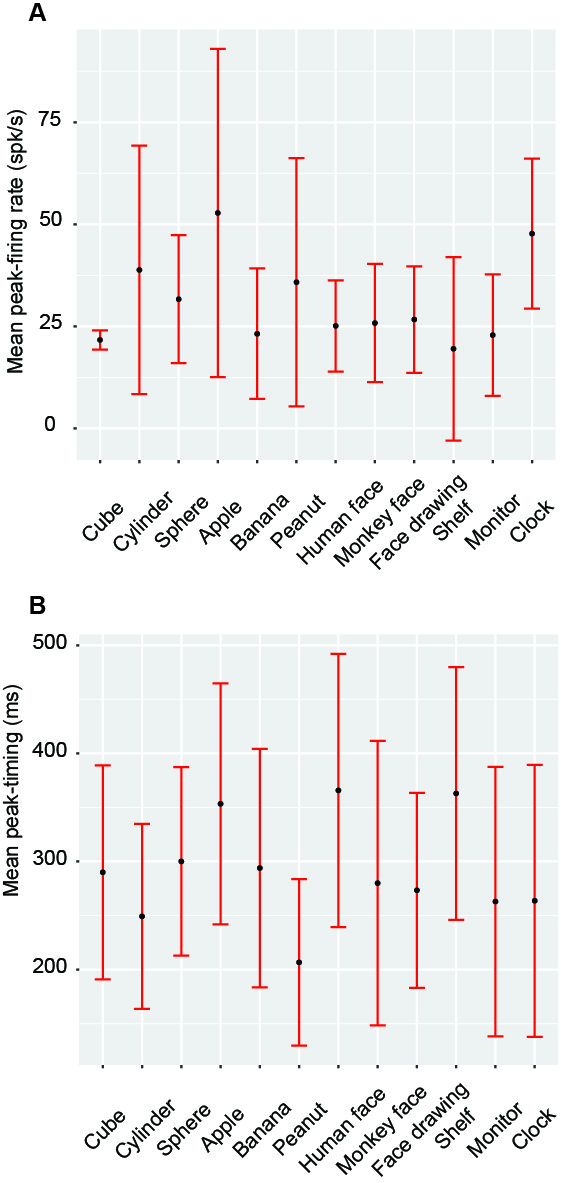


**Supplementary Figure 1**

Average peak-firing rate (a) and average peak timing (b) of neurons coding different stimuli based on their best response (see Table 1 for classification and numerosity). The black dots and the red bars indicate the average and standard deviation of peak (a) and timing (b), respectively. For each neuron, the peak firing rate was identified using a moving average (step=3bins, bin=20ms) of the activity recorded in the Stimulus epoch of the preferred stimulus.
